# A powerful tool for genome analysis in maize: development and evaluation of the high density 600 k SNP genotyping array

**DOI:** 10.1186/1471-2164-15-823

**Published:** 2014-09-29

**Authors:** Sandra Unterseer, Eva Bauer, Georg Haberer, Michael Seidel, Carsten Knaak, Milena Ouzunova, Thomas Meitinger, Tim M Strom, Ruedi Fries, Hubert Pausch, Christofer Bertani, Alessandro Davassi, Klaus FX Mayer, Chris-Carolin Schön

**Affiliations:** Plant Breeding, Centre of Life and Food Sciences Weihenstephan, Technische Universität München, 85354 Freising, Germany; Plant Genome and System Biology, Helmholtz Zentrum München, 85764 Neuherberg, Germany; KWS SAAT AG, 37555 Einbeck, Germany; Institute of Human Genetics, Helmholtz Zentrum München, 85764 Neuherberg, Germany; Animal Breeding, Centre of Life and Food Sciences Weihenstephan, Technische Universität München, 85354 Freising, Germany; Affymetrix Inc., Santa Clara, CA 95051 USA

**Keywords:** High density genotyping array, Maize, SNP

## Abstract

**Background:**

High density genotyping data are indispensable for genomic analyses of complex traits in animal and crop species. Maize is one of the most important crop plants worldwide, however a high density SNP genotyping array for analysis of its large and highly dynamic genome was not available so far.

**Results:**

We developed a high density maize SNP array composed of 616,201 variants (SNPs and small indels). Initially, 57 M variants were discovered by sequencing 30 representative temperate maize lines and then stringently filtered for sequence quality scores and predicted conversion performance on the array resulting in the selection of 1.2 M polymorphic variants assayed on two screening arrays. To identify high-confidence variants, 285 DNA samples from a broad genetic diversity panel of worldwide maize lines including the samples used for sequencing, important founder lines for European maize breeding, hybrids, and proprietary samples with European, US, semi-tropical, and tropical origin were used for experimental validation. We selected 616 k variants according to their performance during validation, support of genotype calls through sequencing data, and physical distribution for further analysis and for the design of the commercially available Affymetrix® Axiom® Maize Genotyping Array. This array is composed of 609,442 SNPs and 6,759 indels. Among these are 116,224 variants in coding regions and 45,655 SNPs of the Illumina® MaizeSNP50 BeadChip for study comparison. In a subset of 45,974 variants, apart from the target SNP additional off-target variants are detected, which show only a minor bias towards intermediate allele frequencies. We performed principal coordinate and admixture analyses to determine the ability of the array to detect and resolve population structure and investigated the extent of LD within a worldwide validation panel.

**Conclusions:**

The high density Affymetrix® Axiom® Maize Genotyping Array is optimized for European and American temperate maize and was developed based on a diverse sample panel by applying stringent quality filter criteria to ensure its suitability for a broad range of applications. With 600 k variants it is the largest currently publically available genotyping array in crop species.

**Electronic supplementary material:**

The online version of this article (doi:10.1186/1471-2164-15-823) contains supplementary material, which is available to authorized users.

## Background

High-throughput genotyping has revolutionized genetic analyses in humans, livestock species, crop and model plants in the past decade [1–3]. Covering genomes with high resolution, single nucleotide polymorphism (SNP) genotyping arrays facilitate the detection of associations between SNPs and phenotypes. They represent a powerful tool for dissecting complex traits via genome-wide association studies (GWAS) or quantitative trait locus (QTL) analysis as well as for fine mapping genes of interest and forward genetics cloning strategies [4–7]. In addition, they are broadly used in crop and livestock breeding for germplasm characterization and marker assisted selection [8]. The availability of high density genotyping arrays has enabled breakthroughs in genome-wide approaches such as genomic prediction and detection of selection signatures [9–12]. Here, we describe the development of the currently largest publicly available SNP array in crop species and discuss its potential for different applications in maize.

Maize is one of the most important crops worldwide serving as food, livestock feed, and component of industrial products. A key step in corn production was the establishment of divergent heterotic patterns for hybrid breeding [13]. Most worldwide hybrid breeding programs exploit heterotic effects between different subgroups within the Dent pool, whereas crosses between the two maize pools, Dent and Flint, are mainly used in hybrid breeding for the cooler regions in Central Europe. Maize production has continuously risen over time, but to further increase selection gain and accelerate breeding processes profound knowledge is required regarding genes and genomic regions involved in agronomically important traits.

Genotyping arrays offer an efficient alternative to whole genome sequence data for gaining genomic information in high-throughput. However, the establishment of a high density genotyping array requires the identification of a large number of variants polymorphic in a representative discovery panel to ensure its utility for a wide range of approaches and study designs. In maize, the identification of sequence variants for genomic analyses faces specific challenges due to its evolutionary history and high variability of its genome. As an ancient polyploid species, the maize genome is characterized by numerous duplicated chromosomal regions giving rise to paralogous sequences [14–16]. A reference sequence exists for maize, which covers around 90% of the 2.4 Gb genome of inbred line B73 (AGP_v2), but the high amount of transposable elements, paralogs, copy number variants (CNV) as well as structural variants like presence/absence variants (PAV), is a challenge for reliable sequence read alignment and variant identification due to ambiguous sequence read mapping results [15, 17, 18]. Despite recent reports like the comprehensive genotyping of the USA national maize inbred seed bank [19] using SNPs identified through genotyping by sequencing (GBS) at low sequence coverage [20], sequencing-based approaches such as GBS have to cope with large amounts of missing data and require the establishment of demanding bioinformatics pipelines and imputing algorithms, which may not be routine in all labs.

The highest resolution of a commercially available genotyping array for maize has been achieved by the Illumina® MaizeSNP50 BeadChip [21]. It has been used extensively for genetic studies [22–25] and is composed of 50 k usable SNPs. This number of SNPs is in the same range as for recently published genotyping arrays for rice [8], soybean [26], and wheat [27], but much lower compared to high density genotyping arrays which are available for animal species, e.g. chicken [28] and cattle with 648 k and 777 k, respectively [29, 30], as well as for humans with more than 900 k SNP variants [5]. Especially for maize with its large genome size and high level of diversity, high marker resolution is desirable. In addition, linkage disequilibrium (LD) decays rapidly in some germplasm, e.g. in landraces or highly diverse sample panels [31] emphazising the requirement of higher marker densities than so far available on genotyping arrays.

We selected sequence variants for the design of a high density 600 k SNP genotyping array for maize based on 57 M SNPs and small indels that were discovered by mapping whole genome sequencing reads of 30 representative temperate maize lines against B73 AGP_v2. For experimental validation, we selected 1.2 M variants by applying stringent filtering criteria. This 1.2 M subset was used to genotype 285 maize samples representing the genetic diversity of European (EU) and American (US) temperate maize as well as a sample of tropical maize lines. We created a final selection of 616,201 high quality variants based on their assay performance, physical distribution, and concordance with *in silico* variant calls from sequencing data. Here, we describe the design of the high density Affymetrix® Axiom® Maize Genotyping Array which represents a powerful tool for fine-mapping of genomic regions, genome-wide studies, and detection of marker-trait associations. We also demonstrate its application for investigating subpopulation structure and LD in diverse maize germplasm.

## Results and discussion

### Discovery and pre-selection of variants

For variant (i.e. SNP and indel) discovery we sequenced 30 maize inbreds composed of 17 European Flint lines as well as nine European and four US Dent lines (Additional file 1: Table S1). The lines represent important founder lines for maize breeding in Europe and the US and have been used in previous studies [32, 33]. Mapping the generated sequence reads to the B73 reference sequence (AGP_v2) resulted in 50-fold sequence coverage on average of four deep sequenced lines (DK105, EP1, F7, PH207) as well as 12-fold coverage on average of the 26 remaining lines. Based on the mapped sequence reads 56,938,462 variant positions were identified.

A filtered list of variants was created for quality score determination similar to the dual approach of Chia et al. [18]. Variants were included in this list if they were identified independently by two different programs, SAMtools [34] and GATK [35] and were characterized by high quality scores as well as presence of reference (B73) and non-reference alleles in the discovery panel. Applying these filters, the initial variant number was reduced by a factor of 10. We finally selected 5,593,169 bi-allelic variants for further analysis. 66.7% (3,731,960) of these variant positions were congruent with variants reported by [18] for the maize HapMap2 data. Of 46,660 variants from the Illumina® MaizeSNP50 BeadChip which could be uniquely anchored to the B73 reference sequence, 43,615 (93.5%) were also covered by *in silico* SNP calls from sequencing in our set of 5.6 M variants. This proportion is higher than the 72.3% overlap reported in the maize HapMap2 SNP dataset reported by [36] and can most likely be attributed to the higher sequence coverage in our study.

### Selection of high-confidence variants for array construction

**Figure 1:**
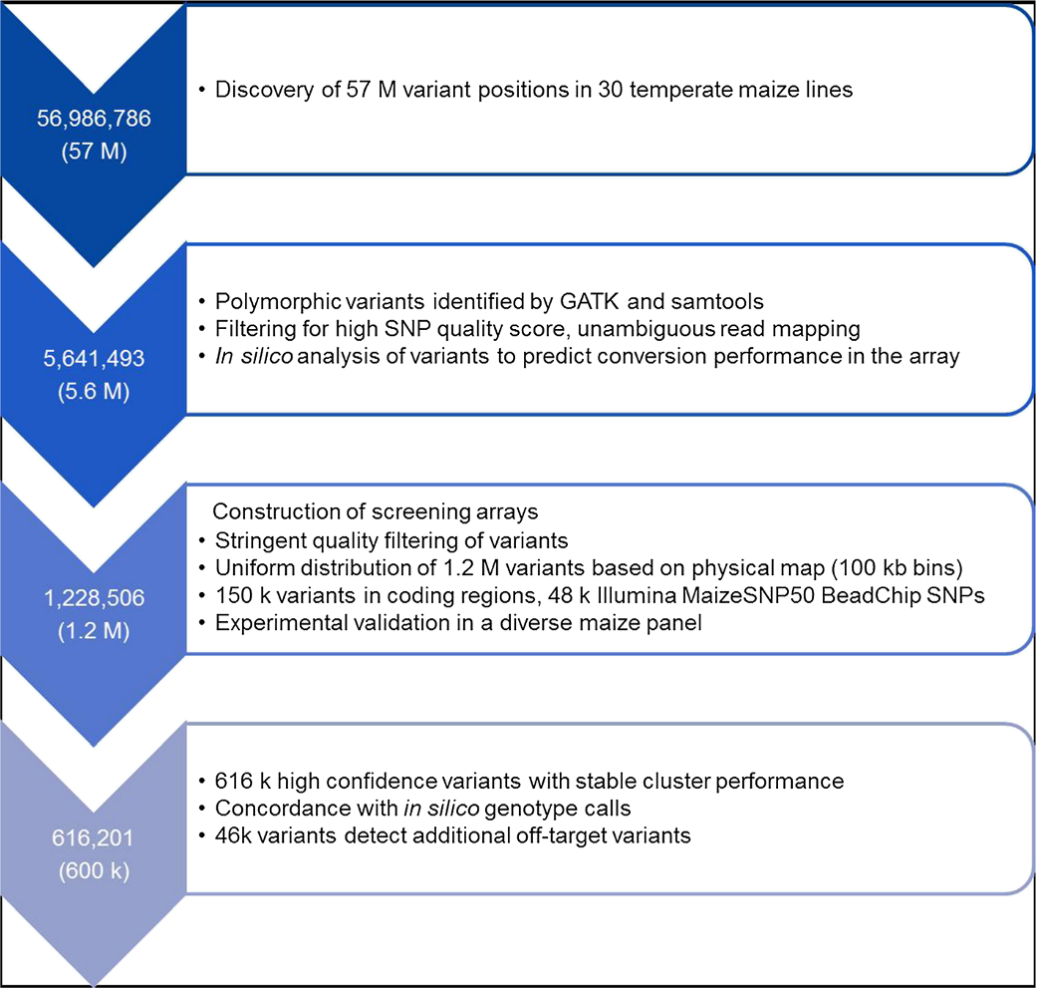
**Flow diagram with the major filtering steps.** Flow diagram showing steps and major criteria of the variant selection process during development of the maize 600 k genotyping array.

A multi-step filtering approach was applied to reduce the number of 5.6 M variants to a subset of 1.2 M variants for experimental validation on two Affymetrix® Axiom® 600 k screening arrays (Figure 1). From those, 616 k were selected for the design of the 600 k array.

#### Variant selection according to in-silico analysis of sequence data

The 5.6 M variants were filtered according to quality and their support by sequence reads. The sequenced lines were inbred lines with only minor residual heterozygosity (mean of 0.65%, Additional file 1: Table S2) as determined from Illumina® MaizeSNP50 data. In the 5.6 M variants, we observed 23.3% heterozygous compared to 72.7% homozygous calls, which was not expected from the Illumina® MaizeSNP50 genotyping data. Besides “true” heterozygous calls, such calls may arise from the large fraction of segmental duplications as well as orthologous and paralogous sequences retained in the ancient polyploid maize genome [15]. In line with this, the false discovery rate (FDR) of heterozygous calls was significantly higher (87.0%) compared to the FDR of homozygous calls (1.6%) as determined by comparison with variant calls from the Illumina® MaizeSNP50 BeadChip. Thus, in order to create a list of high quality variants only homozygous calls were considered for further analysis.

We decided to include all available 150,394 coding variants on the screening arrays, as these variants have a greater potential than non-coding variants to affect gene function. To enable comparison across studies, we further included 48,324 SNPs of the Illumina® MaizeSNP50 BeadChip as “must-have” variants. The remaining ~ 1 M positions on the screening arrays were filled with non-coding variants based on their distribution across the genome. Similar to the strategy reported by Kranis et al. [28], we applied a bin based approach with the intention to create a subset of physically equally distributed variants. We observed that variant numbers in centromeric bins were always lower than in telomeric bins, indicating lower polymorphism rates in the centromeric regions. This reduction of variant numbers around the centromeres was also observed in other maize studies [18, 19, 37] and may result from the high proportion of repetitive DNA around the centromeres for which no markers can be developed. Aiming simultaneously for a balanced representation of pool-specific as well as shared variants between Dent and Flint, 931,340 variants were included in the list for validation. We selected 158,448 additional variants to specifically increase the number of variants in under-represented bins to reach a final number of 1,228,506 variants which could be placed on the screening arrays. The marker density on the screening arrays was one variant per ~ 1.7 kb on average over all chromosomes (Additional file 1: Table S3).

#### Variant validation by genotyping 285 representative maize samples

In order to assemble a robust set of variants for design of the 600 k array, the selected set of 1.2 M variants was used to genotype 285 DNA samples from 280 diverse worldwide maize inbred lines and hybrids for the evaluation of variant performance (Additional file 1: Table S4). We investigated conversion performance of the variants on the array with respect to (i) genotype call rates, cluster separation, and reproducibility, (ii) polymorphism in the panel under study, and (iii) consistent Mendelian inheritance from parents to off-spring in trios.

Hybridization intensity signals were clustered by the Affymetrix Axiom GT1 algorithm and interpreted as homozygous, heterozygous, or no calls, respectively. Different from the situation in humans or animals, where samples are highly heterozygous, most of the samples in our maize validation panel were highly inbred. Thus, we compared genotype calls obtained with and without applying an inbred correction factor (Additional file 2: Figure S1). This factor was assigned to each sample to adjust the probability of observing a heterozygous call given the inbreeding level of the sample. The average call rate of the screening arrays could be increased by 2.3% to 98.1% upon inbred correction (Additional file 1: Table S5). With inbred correction, inbred line B73 exhibited the highest call rate (99.5%) and one F1 hybrid (UH007 x Lo11, 92.2%) together with Teosinte (acc. GID265285, 92.2%) the lowest call rates. Furthermore, American maize lines revealed higher call rates on average compared to European lines, followed by call rates of tropical lines and hybrids. This is in accordance with the literature [21] and suggests a negative correlation between call rate and increasing sequence divergence to the reference sequence of B73 from which probe sequences on the array were derived.

Based on genotype call cluster separation, cluster variance, and cluster position, variants were assigned to one out of six quality categories (Additional file 2: Figure S2). Comparing the category assignments with and without inbred correction resulted in a change of category in 36.2% of all variants (Additional file 1: Table S6). As expected, the category of variants fulfilling all cluster metric criteria and classified as “PolyHighResolution” (PHR) increased most, resulting in a gain of 30.7% upon inbred correction. Details on the number of variants from each category with and without inbred correction are given in Additional file 1: Table S6. In total, 25.1% of the newly developed 1,131,860 variants (excluding the Illumina® MaizeSNP50 variants) failed to convert and did not give reliable genotype calls upon inbred correction (designated “other” in Additional file 1: Table S6). The proportion of 74.9% converted variants is lower than in a similar study in chicken, where 82.0% of the variants could be converted into successful variants [28]. In rice which has an around five-fold smaller and less complex genome than maize, 84% of variants of the Illumina® RiceSNP50 array [8] were converted successfully (GenTrain score > 0.5). Given the higher complexity of the maize genome compared to chicken or rice, our conversion rate is in the expected range.

#### Selection of high-confidence variants and composition of the 600 k array

For the selection of high-confidence variants for the 600 k array, we applied a voting system based on (i) their performance on the screening arrays, (ii) concordance of array genotyping calls with *in silico* variant calls from sequencing data of the 30 maize lines in the discovery panel, and (iii) over- or under-representation of the corresponding bin. To ensure a high performance on the final array, the highest weight was assigned to the first criterion. We focussed on clearly separated genotype clusters with little variance that were not influenced by information regarding the inbreeding level (Additional file 2: Figure S1). Applying this procedure the 570,546 highest scoring variants as well as 45,655 SNPs of the Illumina® MaizeSNP50 BeadChip were included in the final selection for the 600 k array (Additional file 1: Table S6).

**Figure 2:**
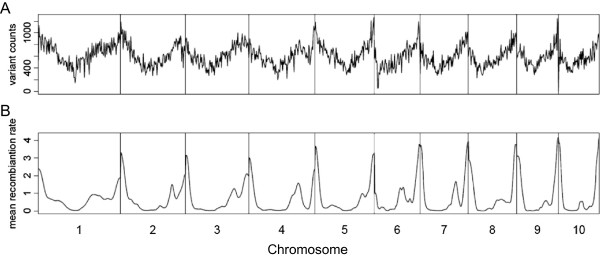
**Physical distribution of 616 k variants and recombination rate.** Physical distribution of variants and average recombination rate along the ten maize chromosomes depicted for 2 Mb windows. **A)** Distribution of 616 k variants represented on the 600 k array, **B)** Average recombination rate in cM/Mb from [32].

The 600 k genotyping array is composed of 616,201 variants (609,442 SNPs and 6,759 indels), corresponding to an average density of one variant per ~ 3.4 kb (median density one variant per 0.3 kb; Additional file 1: Table S3, Additional file 2: Figure S3). The average genetic distance between variants is 0.0025 cM, which corresponds to 406 variants per cM. The variants are evenly distributed across the chromosomes with the only exception of one region on the short arm of chromosome 6, where the maximal distance between neighboring variants exceeds 1.2 Mb. Despite a specific filter aiming for equal variant distribution according to the physical map distance, the final distribution followed the average recombination rate along chromosomes, which reflects varying polymorphism rates in the material under study (Figure 2). The highest density of variants was found in gene enriched telomeric regions, thus ensuring the maximal possible amount of genetic information in regions with high recombination rates. A comparable pattern of variant distribution as well as a lack of variants on the short arm of maize chromosome 6 in the nucleolus organizer region (NOR; approximate position 7–28 Mb) has been reported previously [18, 19]. From the 616,201 variants represented on the Affymetrix® Axiom® Maize Array 561,751 (91.2%) are also present in the maize HapMap2 variants [18].

All 616,201 variant positions were annotated based on the B73 filtered gene set which comprises 39,656 genes (Additional file 1: Table S7), resulting in 26,620 genes (67.1%) tagged with at least one variant in their coding, intronic, or UTR region, compared to 17,520 genes tagged by SNPs of the Illumina® MaizeSNP50 BeadChip (44.2%). Including 5 kb up- and downstream regions, 35,089 genes (88.5%) were represented by at least one variant, thus providing an excellent basis for finding marker-trait associations in targeted and genome-wide approaches.

To determine the reproducibility of variants represented on the 600 k array, technical and biological replicates were analysed. First, three technical B37 replicates as internal controls exhibited up to 99.8% of identical genotype calls (Additional file 1: Table S8). Three biological replicates from different seed sources exhibited a high level of concordant genotype calls in the range of 99.76% to 99.84%. Furthermore, two lines (DK105 and EP1) were represented by two samples each comprised of a single plant and a pooled sample, respectively, showing 99.51% and 97.73% concordance. Some lack of concordance here can be explained by residual heterozygosity in the pooled samples. For determination of stable Mendelian inheritance, 23 trios with both parental lines as well as the corresponding F1 hybrid were analysed. These trios revealed stable Mendelian inheritance between parental lines and their offspring in 94.3% of the variants. After excluding the trio with the lowest call rate (UH007, Lo11, UH007 x Lo11) stable Mendelian inheritance could be observed in 97.6% of the variants, underlining the call rate as an indication of sample quality. The analysis of biological and technical replicates and trios confirmed the high reproducibility of genotype calls obtained with the variants represented on the Affymetrix® Axiom® Maize Array which is in the same range as reported for the Illumina® MaizeSNP50 BeadChip [21].

**Figure 3:**
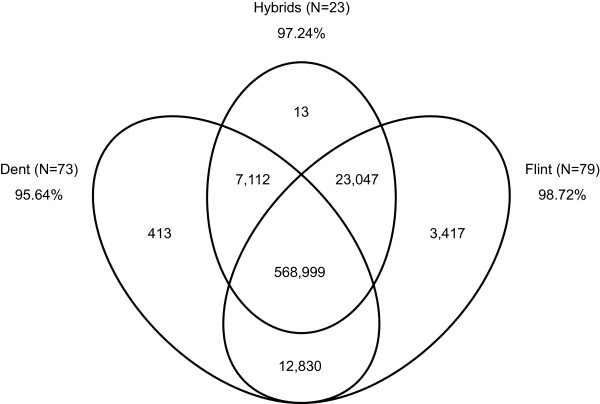
**Polymorphic variants of the 600 k array.** Venn diagram showing the number of polymorphic variants represented on the 600 k array in 73 Dent and 79 Flint samples and 23 hybrids of the validation panel.

The usefulness of a genotyping array is characterized by the number of variants polymorphic in the panel of genotypes under study. In the 155 public maize lines, two Teosinte accessions, and 23 F1 hybrids used in this study for validation, 99.9% of the 600 k array variants were polymorphic. Only a small number of 262 variants (all derived from the Illumina® MaizeSNP50 BeadChip) were monomorphic across all samples of the validation panel. After excluding three genotypic samples without clear germplasm group assignment, 95.6% of the 600 k variants were polymorphic within Dent (N = 73), 98.7% in Flint (N = 79), and 97.2% within F1 hybrids (N = 23), respectively (Figure 3). Only 42.2% of the variants were polymorphic within the two Teosinte accessions. It must be noted however, that with only two samples the diversity in Teosinte is not well captured in our validation panel. Additionally, the array was not optimized for wild maize relatives as they were not included in the discovery panel. The high overall polymorphism rate depicts the quality of the filtering procedure and is in line or even exceeding results obtained by other studies regarding genotype array validation in animals and plants [8, 21, 38, 39]. It confirms the utility of the array for a wide range of applications in maize germplasm.

Among the selected variants, one category called “Off-Target Variants” (OTVs) was of special interest since these 45,974 variants detect previously uncharacterized variants in the flanking region of the target variant. Due to a reduced hybridization efficiency OTVs are characterized by cluster splits or additional relatively low signal intensity clusters compared to expected homozygous and heterozygous genotypes (Additional file 2: Figure S2) and have been shown to be reproducible [40]. These 46 k variants offer the possibility not only to analyse the genotype call of the target variant, but provide in addition information on presence or absence of putative additional variants in the flanking regions. The latter information can be treated as a bi-allelic flanking variant and was included for population structure analyses.

### Analysis of population substructure

**Figure 4:**
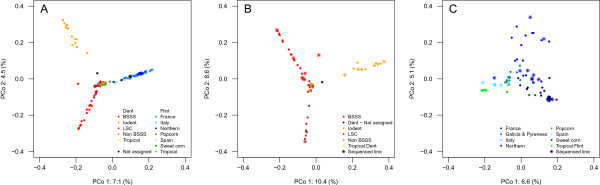
**Population substructure in a diverse maize panel.** PCoA plots of the first two axes in a diverse panel of public maize lines based on Rogers’ distances from 251,152 variants including OTVs represented on the 600 k array (markers in LD with r^2^ > 0.8 were excluded). **A)** Whole set of 155 maize lines, **B)** 73 Dent lines, and **C)** 79 Flint lines.

The identification of population substructure is crucial for quantitative genetic or population genetic studies since population stratification or admixture may affect detection of marker-trait associations, genomic prediction, or estimation of population genetic parameters. To determine the ability of the variants represented on the maize 600 k genotyping array to resolve population structure, we performed principal coordinate (PCoA) and admixture analyses of 155 public inbred lines. The first principal coordinate revealed a clear separation of Dent and Flint with a small group of samples located in the center (Figure 4A). This central group included (sub)tropical Flint and Dent lines, the popcorn accession, two lines with unknown pedigree as well as Flint lines that originated from Southern Spain and one Flint line tracing back to Argentina. The clear separation of Dent and Flint reflects their genetic differentiation for more than 2,500 years [41], accompanied by varying adaptive and selective pressures. Similar results were obtained in studies based on isozymes [42], RFLPs [43, 44], SSRs [41], and SNPs [45]. Analyzing the 73 Dent lines separately (Figure 4B), the first two axes further subdivided the samples into distinct subgroups, namely Iowa Stiff Stalk Synthetic (BSSS), Iodent, and Lancaster Sure Crop (LSC), with several non-BSSS and tropical lines in the center. The three groups BSSS, non-BSSS (including LSC), and Iodent represent three major heterotic groups within temperate Dent, whose strong differentiation is well-known [46, 47]. Compared to US material, European samples clustered within each group more towards the center (Figure 4B), suggesting a lower level of differentiation and population substructure [31].

In Central Europe, Flint plays an important role for hybrid breeding programs relying on the Dent x Flint heterotic pattern. PCoA of 76 Flint lines as well as one popcorn and two sweetcorn accessions resulted in the separation of European Flint lines adapted to more Northern or Mediterranean climate, respectively (Figure 4C). This split has also been observed in other studies based on phenotypic and RFLP marker data [48] and can be traced back to the introgression of maize to Europe. Maize was introduced to Europe starting at the end of the 15^th^ century, when Columbus brought subtropical maize from the Caribbean Islands to Southern Spain, later followed by travelers importing so called Northern Flint [49] from Canada to Northern France [48, 50–52]. The “non-Northern” Flint group in our study was further subdivided in the PCoA by the second axis depicting the relatedness of a subset of samples which had the French line F7 in their ancestry. Thus, the first two axes revealed two main subgroups of European Flint although the substructure was not as pronounced as in Dent. As indicated in Figure 4 (B, C), the sequenced lines of the variant discovery panel were nicely distributed across the different germplasm pools.

Cross-validation results obtained by ADMIXTURE [53] suggested K = 7 as the most likely number of groups (Additional file 2: Figure S4, Additional file 2: Figure S5) with four clear clusters in Dent for BSSS, Iodents, LSC, and a mixed group of non-BSSS lines, as well as two clusters for Northern Flints and non-Northern Flints, and a mixed group of (sub)tropical lines or lines with ancestors of (sub)tropical origin. This grouping well reflects the main subgroups observed with PCoA. In accordance with an increasing sequence divergence to the reference sequence of B73, ADMIXTURE analysis based on the 46 k flanking OTVs resulted in the subdivision of Dent, Flint, and a group including tropical lines as well as Flint lines originating from Argentina, Spain, and Italy (Additional file 2: Figure S6, Additional file 2: Figure S7).

We conclude that the variants represented on the 600 k array are well suited for dissecting the diversity and genetic composition of temperate maize lines. Performance of the array with regard to the analysis of tropical material or wild maize relatives will need further investigation.

### Extent of linkage disequilibrium

The extent of LD in a population is influenced by recombination rate, drift, mutation, selection, and population structure. It has thus influence on experimental design, resolution, and analysis of genome-wide studies. In the public inbred lines genotyped for validation, LD decay (r^2^ ≤ 0.2) could be observed within 158 kb on average with some chromosomal differences (Table 1). Group specific analysis of the LD extent revealed a substantially higher level of LD in the two Dent groups of Iodents and BSSS with mean LD decay distances of 19.5 and 36.2 Mb, respectively, compared to non-BSSS lines (excluding the LSC group) where LD decayed within 239 kb. Due to the rather small sample size in LSC (N = 9), decay distances were not calculated for this subset. Mean LD decay values in Flint were highest for non-Northern Flints, which included several lines sharing a common ancestor, with 4.6 Mb, followed by Northern Flints (312 kb). The fastest LD decay was observed in (sub)tropical lines (70 kb). This corroborates previous reports supporting the close relationship and small number of founder lines within Iodent and BSSS compared to the other groups [19, 47]. The low values of the non-BSSS as well as the (sub)tropical lines in our study might be explained by the high heterogeneity of both groups. Still, LD levels in our panel of maize lines were higher compared to previous studies reporting the breakdown of LD within distances between 5 and 10 kb [18, 54] in highly diverse maize lines. The higher LD extent in our study might be due to the sample panel analysed, which mainly comprised temperate elite maize inbred lines belonging to distinct germplasm pools but no landraces or wild species. The variants selected for the 600 k array fulfill the requirements by [55], after which genotyping arrays should have sufficient coverage to capture the fastest LD decay of the considered heterotic pools. Thus, especially for analysis of diverse sample panels, high density genotyping arrays are of interest for estimating global and local LD.

**Table 1:** **Mean linkage disequilibrium (LD) given as r** ^**2**^ **and average LD decay distance** ^**a**^ **in kb per chromosome in 155 lines (all) and in six** ^**b**^ **subgroups as determined by ADMIXTURE**

	All		Dent – BSSS		Dent – Non-BSSS		Iodent		Non-Northern Flint		Northern Flint		(Sub) Tropical	
	(N = 155)		(N = 14)		(N = 32)		(N = 14)		(N = 18)		(N = 34)		(N = 34)	
Chr.	mean r ^2^	r ^2^ decay	mean r ^2^	r ^2^ decay	mean r ^2^	r ^2^ decay	mean r ^2^	r ^2^ decay	mean r ^2^	r ^2^ decay	mean r ^2^	r ^2^ decay	mean r ^2^	r ^2^ decay
1	0.029	119.14	0.202	14,411.28	0.049	156.32	0.212	15,949.23	0.133	4,007.83	0.049	197.98	0.037	43.38
2	0.027	126.13	0.260	30,329.28	0.048	178.63	0.177	9,411.84	0.133	4,211.89	0.059	332.88	0.039	64.29
3	0.034	199.62	0.263	31,772.64	0.057	306.29	0.170	8,340.20	0.199	15,545.48	0.055	352.29	0.044	96.22
4	0.031	192.41	0.207	16,123.82	0.054	319.43	0.310	50,389.31	0.128	3,114.28	0.063	404.96	0.037	83.82
5	0.027	119.91	0.224	18,692.30	0.047	172.69	0.154	5,681.66	0.144	4,626.09	0.056	262.24	0.038	57.22
6	0.025	106.65	0.198	14,001.18	0.049	188.22	0.170	8,886.16	0.099	955.38	0.049	206.58	0.036	40.20
7	0.033	176.04	0.214	16,901.24	0.057	246.89	0.228	18,945.94	0.141	3,737.87	0.060	379.57	0.042	75.11
8	0.033	183.72	0.257	28,937.29	0.053	254.27	0.280	37,984.38	0.120	1,537.13	0.052	322.23	0.039	81.56
9	0.033	167.58	0.309	53,758.98	0.057	263.68	0.263	28,009.93	0.151	5,020.05	0.056	314.73	0.041	61.48
10	0.033	192.64	0.396	136,831.20	0.057	303.38	0.256	11,899.95	0.130	2,916.32	0.054	343.07	0.041	96.65
Mean	0.031	158.38	0.253	36,175.92	0.053	238.98	0.222	19,549.86	0.138	4,567.23	0.055	311.65	0.039	69.99
Median	0.032	171.81	0.241	23,814.80	0.054	250.58	0.220	13,924.59	0.133	3,872.85	0.055	327.55	0.039	69.70

^a^Distances in kb for r^2^ = 0.2 calculated per 50 Mb window.

^b^LD decay distances were not calculated for LSC (N = 9).

### Other potential applications of the maize high density array

**Figure 5:**
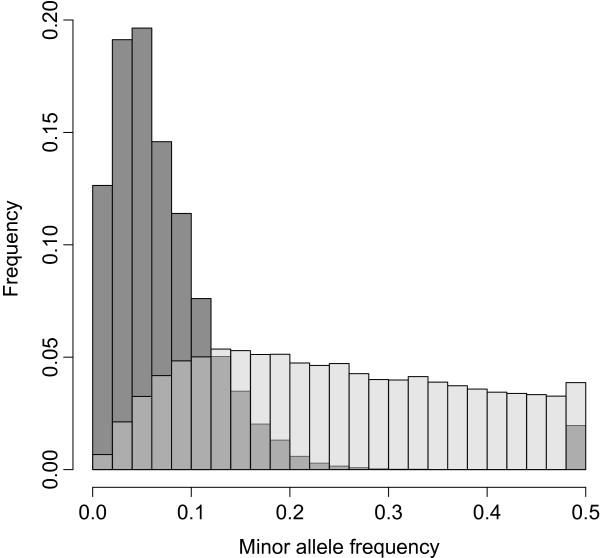
**Minor allele frequency distribution for 616 k variants and 46 k flanking OTVs.** Minor allele frequency (MAF) distribution in 155 maize lines for the 616 k variants (transparent grey) and for 46 k flanking OTVs (black).

We presented the usefulness of the maize high density 600 k array for the analysis of population structure and LD, but of course it is suitable for many other applications in maize research and breeding. For population genetic analyses based on genotyping data ascertainment bias is a central aspect [56]. The lines sequenced in this study were chosen to represent the diversity within the more comprehensive validation panel. However, bias may be introduced by the filtering steps that are applied during array development and typically results in a bias towards intermediate allele frequencies. Flanking OTVs have the advantage of not being directly targeted by the variant filtering procedure itself offering thus the potential to counteract ascertainment bias [40]. Compared to the minor allele frequency (MAF) distribution of the complete set of variants of the 600 k array where only 3.8% of the variants detect rare alleles with a MAF < 0.05, OTVs showed with 40.8% rare alleles (MAF < 0.05) a reduced bias towards intermediate allele frequencies (Figure 5). Thus, even if the specific type of the variant as well as its exact location is unknown, flanking OTVs represent an interesting subset of variants for population genetic analyses like screens for selection signatures based on genotyping data which we will address in further studies.

Further applications of the 600 k array include its use in genome-wide and targeted approaches. The array should have the desired density for genome-wide association studies in maize, for which the currently available density of the Illumina® MaizeSNP50 BeadChip was shown to provide limited resolution [57]. Due to the extremely high marker density, the array can be used for bulked segregant analysis to identify genomic regions involved in phenotypic traits with monogenic or oligogenic inheritance [58]. Further applications may be seen in the context of plant variety protection and in the investigation of pedigree relationships, identity-by-descent regions and ancestral lineages [59]. A panel of representative lines genotyped with the 600 k array should also allow high accuracy in imputation of genotypes from genetic material analysed with lower density marker panels [60]. Finally, custom sets of SNPs may be assembled from any genomic region and converted into other highly flexible SNP assay formats to saturate specific regions in fine-mapping, map-based cloning studies or marker-assisted selection, since flanking sequence information is available and conversion rates among SNP platforms are generally high.

## Conclusions

This paper describes the establishment of the currently largest publicly available SNP array in crop species composed of 616,201 SNPs and small indels. The Affymetrix® Axiom® Maize Array is optimized for European and American temperate maize. It is well suited for fine-mapping of genomic regions, genome-wide studies, and detection of marker-trait associations. Important aspects in the development of the maize 600 k genotyping array were: (i) identification of polymorphic, high-confidence variants based on whole genome sequence data of 30 representative temperate maize lines, (ii) selection of physically equally distributed variants for validation, taking predicted variant performance and subgroup specific segregation into account, (iii) experimental variant validation by genotyping 285 DNA samples originating from diverse subgroups of maize, and (iv) final selection of variants upon stringent filtering based on cluster metrics, concordance with *in silico* calls, and physical position. We have shown that the variants selected for the 600 k genotyping array were polymorphic in a broad maize panel, ensuring its suitability for a wide range of applications. We investigated a subset of variants (OTVs) that showed almost no bias towards intermediate allele frequencies, thus are potentially of interest for population genetic analyses. Finally, we performed principal coordinate, admixture, and LD analyses to illustrate the potential of the array to analyse population substructure and LD decay with high resolution.

## Methods

### Sequencing of 30 maize lines in the variant discovery panel

For whole genome sequencing of 30 maize inbred lines (Additional file 1: Table S1), DNA was extracted from leaf material frozen in liquid nitrogen following the protocol of [61]. For deep-sequencing of the three Flint lines DK105, EP1, and F7, as well as the Dent line PH207, DNA was extracted from a single plant each. For sequencing of the other 26 maize lines, DNA was extracted from bulked leaf samples of 8–10 plants per line. Sequencing was performed on an Illumina® HiSeq 2000 platform, generating 2x100 bp paired-end reads from standard 300 bp libraries using manufacturer’s protocols.

### *In silico*variant discovery and pre-selection

Sequence reads were mapped against reference sequence B73 AGP_v2 using BWA (version 0.7.5) [62]. Alignments were post-processed by marking duplicates and fixing paired-end information applying the PICARD toolbox (version 1.84) [63] and by performing local realignments using the Genome Analysis Toolkit (GATK, version 2.4-9) [35]. Quality scores of called variant positions (SNPs and short insertions/deletions) were improved by recalibrating values according to results of several covariate analyses (homopolymer, cycle, dinucleotide, quality score) done on a set of trusted variants. As no SNP database was available for the maize varieties under study, a database of high quality trusted SNPs was created following the recommendation on the GATK website (http://www.broadinstitute.org/gatk/guide/tagged?tag=baserecalibrator).

Briefly, initial variants were called independently using two algorithms to obtain a more robust SNP set, as recommended by [64]. We used SAMtools (version 0.1.18) [34] and the intersection to the initial GATK variants was further filtered for SNP quality (≥ 50); low and excessive read coverage (50 ≤ DP < 3000); presence of the reference allele; and homozygous non-reference calls in at least two of the 30 lines. In a second round, variants were identified from the base quality recalibrated bam files by the GATK Unified Genotyper. For the final set of candidate variants, several stringent filters were applied. First, variants were excluded if they were located in regions with genomic copy number ≥ 50 (based on 16-kmer counts). Second, variants were not forwarded to the next step if (i) more than 5% of reads had mapping quality 0, (ii) coverage was more than six fold higher compared to the mean coverage, or (iii) a SNP quality score was below 100. In addition, variants had to exhibit a minimal distance of 20 bp between neighboring variants located in at least one flanking sequence. In summary, the final pre-selection variant set scored for tiling by the Affymetrix® Axiom® myDesign GW bioinformatics pipeline comprised a total of 5,641,493 bi-allelic variants. For annotation of the variants, version 5b60 of the reference sequence B73 AGP_v2 was used (ftp://ftp.gramene.org/pub/gramene/maizesequence.org/release-5b/filtered-set/) which contains 39,656 gene models. Variant effects were predicted using SNPeff (version 3.2) [65].

### Variant selection for the screening arrays according to predicted conversion quality, physical position, and segregation in Dent and Flint

For all variants from the 5.6 M list p-convert values were calculated per probe according to the Affymetrix® Axiom myDesign GW bioinformatics pipeline and categorized as “recommended”, “neutral”, “not recommended”, and “not possible”, respectively. The p-convert value can take a value between 0 and 1 and describes the predicted probability to convert on the array by taking its sequence, binding energies, expected degree of non-specific binding and hybridization to multiple genomic regions into account. Two probe sets (forward and reverse) for each SNP from the Illumina® MaizeSNP50 BeadChip (GenTrain score > 0) were directly included in the list of variants for the screening arrays without further filtering unless they were classified as “not possible”. For the newly identified variants only probe sets categorized as “recommended” or “neutral” were further analyzed. For coding variants, the probe with the higher p-convert value was chosen based on this classification, whereas for all remaining variants probe sets were further filtered according to the following multi step approach. Based on the reference genome size of 2.066 Gb, first, the maize genome was partitioned in 20,660 bins of size 100 kb, aiming at an equal physical distribution of variants. Assuming up to 1.23 M possible variants, which could be tested on two screening arrays, after substracting the fixed variants (150 k coding variants and 2*48 k Illumina® MaizeSNP50 BeadChip SNPs), each 100 kb bin would contain on average 48 variants. Three cases were distinguished to fill the physical bins: (i) all possible variants of a bin were included if less than 48 “recommended” or “neutral” variants were identified in the corresponding bin, (ii) “recommended” variants were considered as fixed, if their number did not exceed 48 and remaining “neutral” variants were subjected to another filtering step, and (iii) “recommended” variants were further filtered, if ≥ 48 were observed in the corresponding bin. For this filtering step to fill up underrepresented bins, allele frequencies were determined for Dent (N = 13) and Flint (N = 17) lines separately by calculating the ratio of homozygous non-reference allele calls in relation to all available calls per variant. Variants were classified according to their pool-specific allele frequency as class “A”, corresponding to intermediate (between 0.2 and 0.8), or “B” to extreme non-reference allele frequencies (< 0.2 or > 0.8). One third of the variants, which filled up the bins, were chosen to be specific for Flint (category Dent “A” | Flint “B”) and one third specific for Dent (Dent “B” | Flint “A”), respectively. Further, one sixth each had to be either common (category Dent “A” | Flint “A”) or rare for both groups (Dent “B” | Flint “B”), respectively.

In a final step, 50 kb bins were considered if the original 100 kb bin had been filled with less than 48 variants. Additional variants were selected if there were less than 8 variants per 50 kb bin to avoid underrepresentation of genomic regions by choosing variants randomly (i) from “recommended” variants with at least six, but less than 22 homozygous reference allele calls in at least 28 of the 30 lines of the discovery panel to avoid extreme allele frequencies (maximal six variants per 50 kb bin), and (ii) if further variants were required, from all remaining variants. Altogether, a final list of 1,228,506 variants was established for validation with a diverse panel of maize lines on two customized 675 k Affymetrix® Axiom® myDesign GW screening arrays.

### Plant material for genotyping

The selected set of 1.2 M variants was used to genotype 285 DNA samples from genetically diverse maize germplasm to evaluate their assay performance. The validation panel was composed of 224 Dent and Flint inbred lines of which 92 were proprietary lines. From those, line B37 was included three times as technical replicate and three lines (B73, DK105, EP1) were represented by two biological replicates each. In addition, 13 tropical lines (ten Flint, three Dent), ten doubled haploid lines from three European Flint landraces, four lines with no available pool assignment, and two Teosinte accessions were analysed. Finally, we included 27 hybrids, among which there were 23 F1 hybrids from Mendelian trios with both parental lines present in the public elite line panel, and four proprietary hybrids (Additional file 1: Table S4). The Dent elite lines comprised representative samples belonging to the subgroups Iowa Stiff Stalk Synthetic (BSSS), Lancaster Sure Crop (LSC), or Iodent, as well as other non-BSSS samples, and samples with tropical origin. The Flint panel was composed of European Northern Flints and lines originating from Spain, Italy, and France, as well as sweetcorn, popcorn, and tropical lines. Except for the 92 proprietary inbred lines, the elite inbred lines were selected according to their frequency of use and citation [46, 47] as well as based on utilization in other studies, pedigree information or classifcation available from literature [66, 67] or from internet sources [68] with the aim to represent diverse temperate material. The 96 proprietary samples were included in the analysis of the screening array for training of the variant clustering algorithm, but not in further analyses presented here.

DNA for genotyping was extracted from seeds available to the authors or kindly provided by the following institutions: INRA UMR de Génétique Végétale (Gif-sur-Yvette, France), Universität Hohenheim (Stuttgart, Germany), USDA-ARS (Ames, USA), CIAM (La Coruña, Spain), CRA-MAC Maize Research Unit (Bergamo, Italy), and CSIC (Pontevedra, Spain).

### Comparison of variant calls with the Illumina® MaizeSNP50 BeadChip

The 30 sequenced lines (Additional file 1: Table S1) were genotyped with the Illumina® MaizeSNP50 BeadChip following manufacturer’s protocols using a total of 50 ng genomic DNA. Raw hybridization intensity data processing, clustering, and genotype calling were performed using the software GenomeStudio (v2011.1, Illumina®) and the public cluster file II described in [21].

### Experimental variant validation by genotyping

From each sample, 200 ng genomic DNA per array was used for analysis on the Affymetrix GeneTitan® platform with the Axiom myDesign GW genotyping array following manufacturer’s protocol. After array processing, four samples were excluded from further analyses as signal intensity files could be created for only one of the two screening arrays, resulting in 281 samples remaining for further investigation (Additional file 1: Table S4).

Raw hybridization intensity data processing, clustering, genotype calling (genotypes AA, AB, BB), off-target variant (OTV; genotypes AA, AB, BB, OO) calling, and variant categorization according to genotype cluster metrics (Additional file 2: Figure S2) were performed using Affymetrix Power Tools (APT, version 1.15.0) and the package SNPolisher (version 1.3.6.6) [69] for R (version 3.0.1) [70] according to the Axiom Genotyping Solution Data Analysis Guide. For initial genotype calling generic *a priori* cluster positions were used since no information about expected cluster positions was available. The three possible genotype clusters were then redefined in *a posteriori* cluster positions, taking the observed genotype call positions into account and variants were finally classified according to selected cluster metrics. A first analysis was performed according to the recommendations of Affymetrix, but with a reduced threshold (0.90) for the variant call frequency instead of the default value (0.97) to account for the high amount of PAVs in the maize genome [17].

In a second, extended analysis different levels of inbreeding were taken into account for *a posteriori* cluster definition because of the high amount of lines in the validation panel exhibiting only a small proportion of heterozygosity in contrast to populations in Hardy-Weinberg equilibrium. The inbred correction was achieved by a parameter assigning sample-specific penalties using the “−read-inbred” parameter for the “apt-probeset-genotype” command in APT. This parameter takes values from 0 for fully heterozygous to 16 for completely homozygous samples and includes this information for re-defining *a priori* cluster positions for genotype calling. We assigned values of 0 for F1 hybrids, 12 for inbreds with unclear homozygosity level, and 14 for pure inbred and doubled haploid lines to allow some remaining heterozygosity (Additional file 1: Table S4). Results of the analyses with and without inbred correction were compared and a subset of randomly selected genotype clusters were visually checked.

### Selection of high-confidence variants for construction of the final 600 k array

Variants were preferentially selected if they were exhibiting stable category assignments (Additional file 2: Figure S2) with clearly separated clusters to avoid restrictions dependent on the inbred-level. Categories were assigned by the classification step of SNPolisher using the following parameters: CR.cut = 90, FLD.cut = 3.6, HetSO.cut = −0.1, HetSO.OTV.cut = −0.3, HomRO2.cut = 0.3, HomRO3.cut = −0.9, HomRO.flag = TRUE, nMinorAllele.cut = 2. For high quality variant selection, a total of 523,154 variants classified as “PolyHighResolution” (PHR) with and without inbred correction were directly forwarded to the final list as they were characterized by distinct and narrow clusters in both analyses. These variants were used to define customized cluster quality criteria for OTVs to ensure a clear separation of genotype clusters, but allowing in addition lower heterozygous cluster signal intensities due to cluster splits caused by unexpected off-target variants in the flanking region of the target variant or potential tri-allelic variants. The “Fisher´s Linear Discriminant” (FLD) value characterized the cluster quality being highest in case of well-separated and narrow clusters. The “Heterozygous Cluster Strength Offset” (HetSO) measured the difference in the signal intensities of the genotype clusters as the heterozygous cluster should have higher signal intensity on average compared to the homozygous ones due to technical features of the array. The “Homozygote Ratio Offset” (HomRO) described the distance of the homozygous clusters to the heterozygous one to detect potentially misplaced clusters. The chosen thresholds upon inbred correction were the following: no monomorphic variants, ≤ 10% missing calls (corresponding to ≤ 30 missing calls), FLD > 3.5, HetSO > −3.5, and HomRO > 1. As FLD and HetSO values were exhibiting missing values in some variants with only two clusters, an additional threshold was set in this case using a FLD value between homozygous clusters (homFLD) of > 5. All 42,877 variants which were classified in both analyses (with and without inbred correction) as OTV and passed in addition the above thresholds were included in the selection for the final array.

Remaining variants were ranked by applying a voting system. First, variants were ranked according to their classification with and without inbred correction. Variants, which were classified as “OTVstable” or changed their category from “NoMinorHom” (only one homozygous and one heterozygous genotype cluster) without inbred correction to PHR after inbred correction, were assigned a weight of 10. Variants, which belonged to any other class without inbred correction, but changed to PHR after inbred correction received a weight of 5, and all remaining variants not fulfilling the previous criteria obtained a weight of 0. Second, variants were weighted regarding the concordance of their calls with the *in silico* variant calls from sequencing. The number of matching calls per variant across the 30 sequenced lines from the discovery panel, which all were also analyzed on the genotyping test arrays, was normalized to the total number of calls per variant resulting in a value in the range of 0 to 1. As a third criterion, the over- or underrepresentation of the corresponding 100 kb bin was taken into account by calculating the deviation of the number of variants in the corresponding bin to the mean of variants in the five bins up- and downstream, respectively, and scaling the value into a range of values between −1 and 1. For the final rank, the sum was built of (i) the weight of the variant class that was multiplied with 35 to ensure a high performance on the final array (range: 0 to 750), (ii) the value of the sequence match multiplied with 90 to minimize false-positives (range: 0 to 90), and (iii) the weight of the bin representation, which was multiplied by 10 for lowest impact (range: −10 to 10). For the 48,324 Illumina® MaizeSNP50 SNPs which were tiled from both sides, the probe with the higher rank was included in the final set in case of varying ranks. If both probes of a variant exhibited the same rank, one probe was chosen randomly. Due to an erroneous mapping of 2,669 Illumina® MaizeSNP50 BeadChip SNPs to the B73 reference sequence a wrong (non-polymorphic) position was assayed on the screening arrays and these non-validated SNPs were not included on the final array. The top 616,201 variants were selected for the final array design among which 45,655 originated from the Illumina® MaizeSNP50 BeadChip. Information on SNP IDs, genome positions, probe sets, and alleles are available at NCBI GEO as platform GPL18778 (http://www.ncbi.nlm.nih.gov/geo/query/acc.cgi?acc=GPL18778) or from the product information of manufacturer Affymetrix.

### Analyses of population substructure and linkage disequilibrium

For all analyses indels were treated as bi-allelic SNPs. In PCoA and ADMIXTURE analyses OTVs were included with their genotype calls of the target variant as well as information on presence or absence of a flanking variant, resulting in 616 k plus 46 k variants. Variants with ≥ 10% of missing data were excluded. Remaining missing data were imputed using Beagle [71] via the R package “synbreed” [72] with R version 3.0.1 [70]. Public inbreds (N = 155, replicates excluded) of the validation panel were investigated with PCoA and ADMIXTURE for population structure as well as for LD decay. LD pruning was performed for PCoA and ADMIXTURE analyses by applying a r^2^ threshold of 0.8. PCoA based on Rogers´ distances was performed using R with the packages “synbreed” [72], “adegenet” [73], and “ape” [74]. Analysis of population substructure was calculated using ADMIXTURE (version 1.23) [53] running with default settings for K = 1 to K = 15. LD was calculated chromosome-wise per 50 Mb window using Plink (version 1.07) [75] and LD decay analysis was performed using the R package “synbreed” [72].

## Availability of supporting data

Supporting sequence data are available in the NCBI Sequence Read Archive (SRA) repository under BioProject accession number PRJNA260788 (http://www.ncbi.nlm.nih.gov/bioproject/PRJNA260788). Information on SNP IDs, genome positions, probe sets, and alleles can be retrieved from NCBI GEO, platform GPL18778 (http://www.ncbi.nlm.nih.gov/geo/query/acc.cgi?acc=GPL18778).

## Electronic supplementary material

Additional file 1: Table S1: Description of the sequence variant discovery panel with group assignment, origin (Europe or USA), raw sequence coverage, number of replicates per line, and assigned inbred penalty (cf. genotype calling in Material and Methods section). **Table S2.** Percentage of heterozygous and missing calls, respectively, for samples of the discovery panel calculated from 49,574 Illumina® MaizeSNP50 BeadChip SNPs with a GenTrain score > 0. **Table S3.** Number of variants as well as median, mean, and maximal distance between neighboring variants in kb per chromosome and mean genetic distance in cM for screening arrays and final array, respectively. **Table S4.** Description of validation panel with group assignment, origin (CA: Canada, EU: Europe, MX: Mexico, SA: South Africa, US: United States of America, “-“: no information available), source of the material (proprietary: plant material from KWS SAAT AG), inbred penalty, and number of replicates. **Table S5.** Call rates of validation samples (N = 281) on the two screening arrays, without and with inbred correction. Samples are sorted according to mean call rate with inbred correction across arrays (last column). **Table S6.** Number and percentage of variants per category with and without inbred correction for new identified and Illumina® MaizeSNP50 BeadChip variants, respectively, on the screening arrays and on the 600 k array. **Table S7.** Annotation and prediction of variant effects for the 616,201 variants of the maize 600 k array. Predictions were obtained with SNPeff [65]. Multiple entries per variant are possible. **Table S8.** Overview of replicates included in the validation panel and corresponding percentage of genotype call concordance calculcated from 570 k SNPs (omitting variants with flanking OTVs). (XLSX 96 KB)

Additional file 2: Figure S1: Effects of inbred correction on genotype calling in predominantly homozygous inbred lines shown for two variants. **Figure S2.** Representative cluster plots for the six categories according to SNPolisher. **Figure S3.** Variant density shown for the screening arrays (light grey) and for the variants of the Affymetrix® Axiom® Maize Array (black) across the 10 maize chromosomes. Centromere positions are indicated by a black horizontal bar. **Figure S4.** Cross-validation errors from ADMIXTURE for different values of K for 155 maize lines based on 251,152 variants including OTVs (markers in LD with r^2^ > 0.8 were excluded). **Figure S5.** Subgroups identified in 155 maize lines of the validation panel as revealed by ADMIXTURE for K = 7 based on 251,152 variants including OTVs (markers in LD with r^2^ > 0.8 were excluded). **Figure S6.** Cross-validation errors from ADMIXTURE for different values of K for 155 maize lines based on 27,099 flanking OTVs (markers in LD with r^2^ > 0.8 were excluded). **Figure S7.** Subgroups identified in the 155 public lines of the validation panel as revealed by admixture for K = 3 based on 27,099 flanking OTVs (markers in LD with r^2^ > 0.8 were excluded). (PDF 1 MB)
