# Supplementary material for: A powerful tool for genome analysis in maize: development and evaluation of the high density 600 k SNP genotyping array
Source: BMC Genomics. 2014 Sep 29;15(1):823. doi: 10.1186/1471-2164-15-823 (PMC4192734; doi:10.1186/1471-2164-15-823)
Supplement: Supplementary file 2 — Additional file 2: Figure S1: Effects of inbred correction on genotype calling in predominantly homozygous inbred lines shown for two variants. Figure S2. Representative cluster plots for the six categories according to SNPolisher. Figure S3. Variant density shown for the screening arrays (light grey) and for the variants of the Affymetrix® Axiom® Maize Array (black) across the 10 maize chromosomes. Centromere positions are indicated by a black horizontal bar. Figure S4. Cross-validation errors from ADMIXTURE for different values of K for 155 maize lines based on 251,152 variants including OTVs (markers in LD with r2 > 0.8 were excluded). Figure S5. Subgroups identified in 155 maize lines of the validation panel as revealed by ADMIXTURE for K = 7 based on 251,152 variants including OTVs (markers in LD with r2 > 0.8 were excluded). Figure S6. Cross-validation errors from ADMIXTURE for different values of K for 155 maize lines based on 27,099 flanking OTVs (markers in LD with r2 > 0.8 were excluded). Figure S7. Subgroups identified in the 155 public lines of the validation panel as revealed by admixture for K = 3 based on 27,099 flanking OTVs (markers in LD with r2 > 0.8 were excluded). (PDF 1 MB) [file 12864_2014_6516_MOESM2_ESM.pdf]

Supplemental Figures

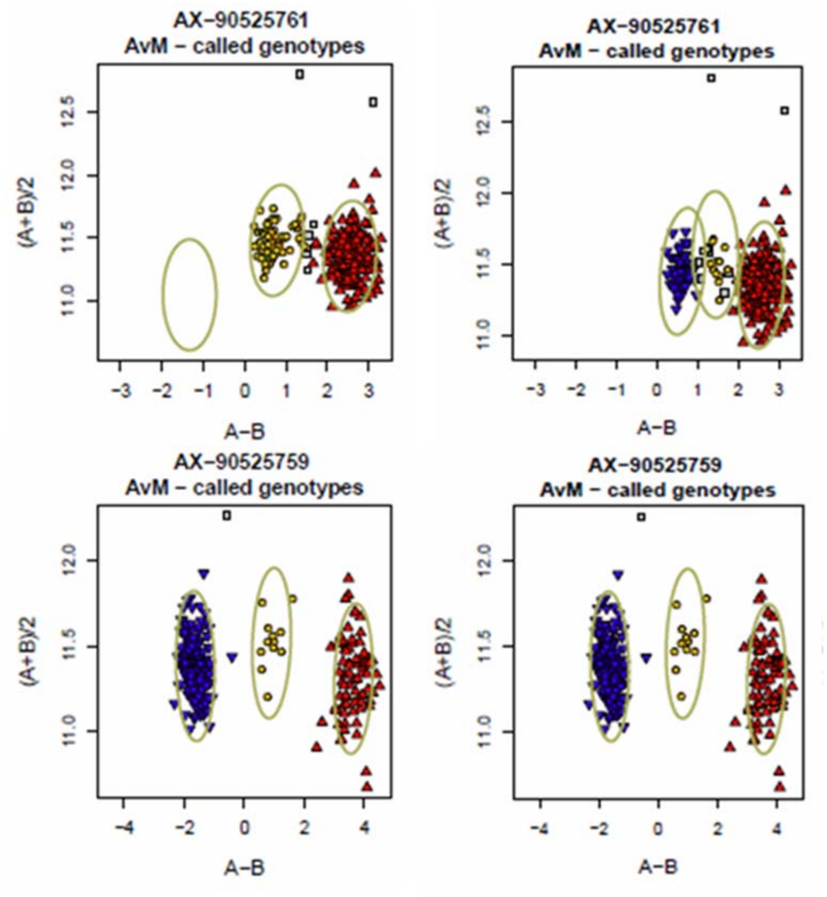

Figure S1: Effects of inbred correction on genotype calling in predominantly homozygous inbred lines shown for two variants.

For marker AX-90525761 (top), the cluster positions change after inbred correction, whereas for marker AX-90525759 (bottom) cluster positions are stable. Left: without inbred correction, right: with inbred correction; X-axis: contrast of the two color channels for allele A and B, respectively, Y-axis: signal intensity; blue circles: homozygous for allele A, yellow triangles: heterozygous genotypes, red circles: homozygous for allele B, grey rectangles: no calls.

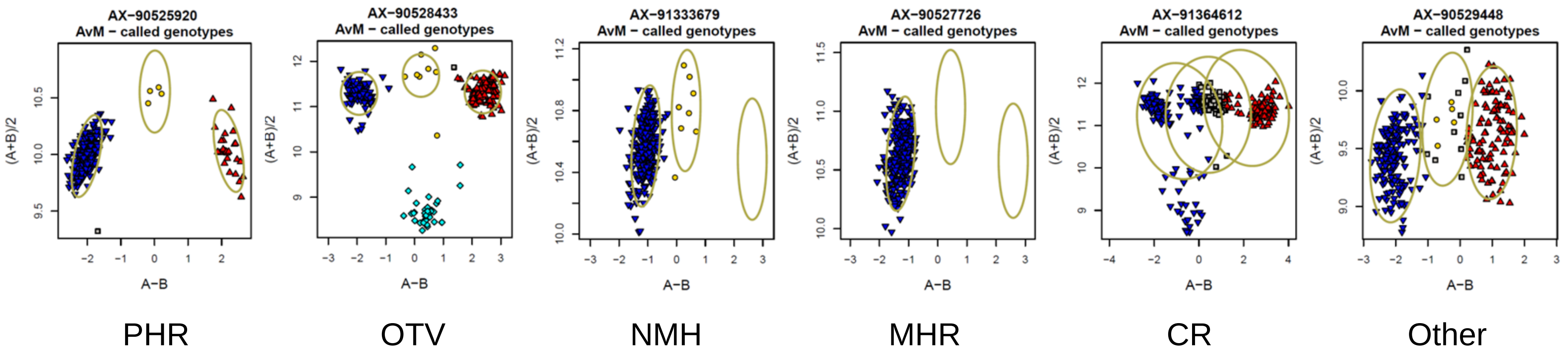

Figure S2: Representative cluster plots for the six categories according to SNPolisher.

From left to right: PHR - “PolyHighResolution“ with three clearly separated and called clusters; OTV - “Off-Target Variant“ indicated by a potential cluster split and/or low signal intensity calls; NMH - “NoMinorHom” with one homozygous cluster without genotype calls; MHR - “MonoHighResolution“ with calls belonging to one genotype cluster only; CR - “CallRateBelowThreshold“ associated with overlapping and mis-assigned clusters; Other - several criteria not fulfilled.

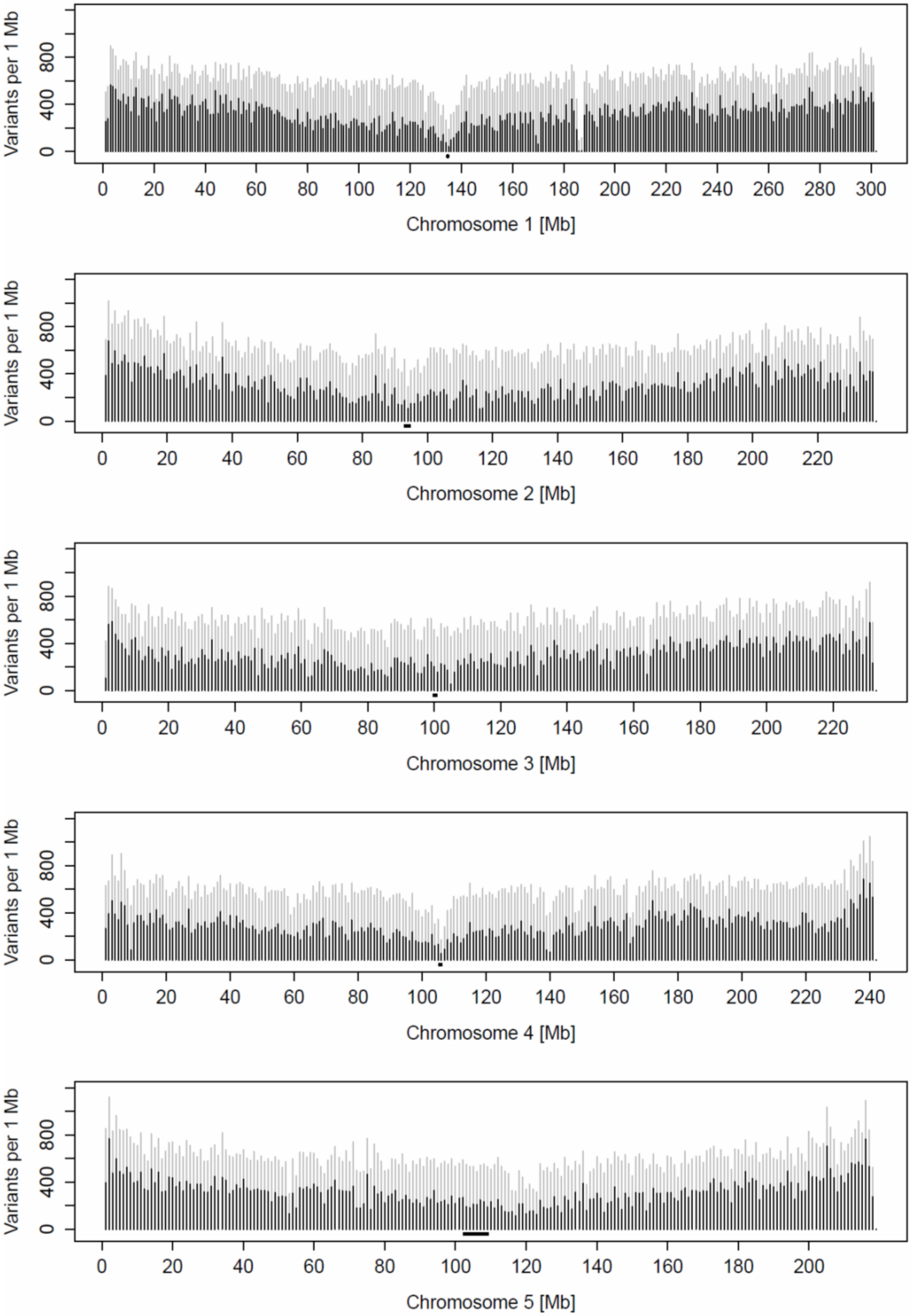

Figure S3: Variant density shown for the screening arrays (light grey) and for the 616 k variants of the Affymetrix® Axiom® Maize Array (black) across the 10 maize chromosomes. Centromere positions are indicated by a black horizontal bar.

Figure S3 continued

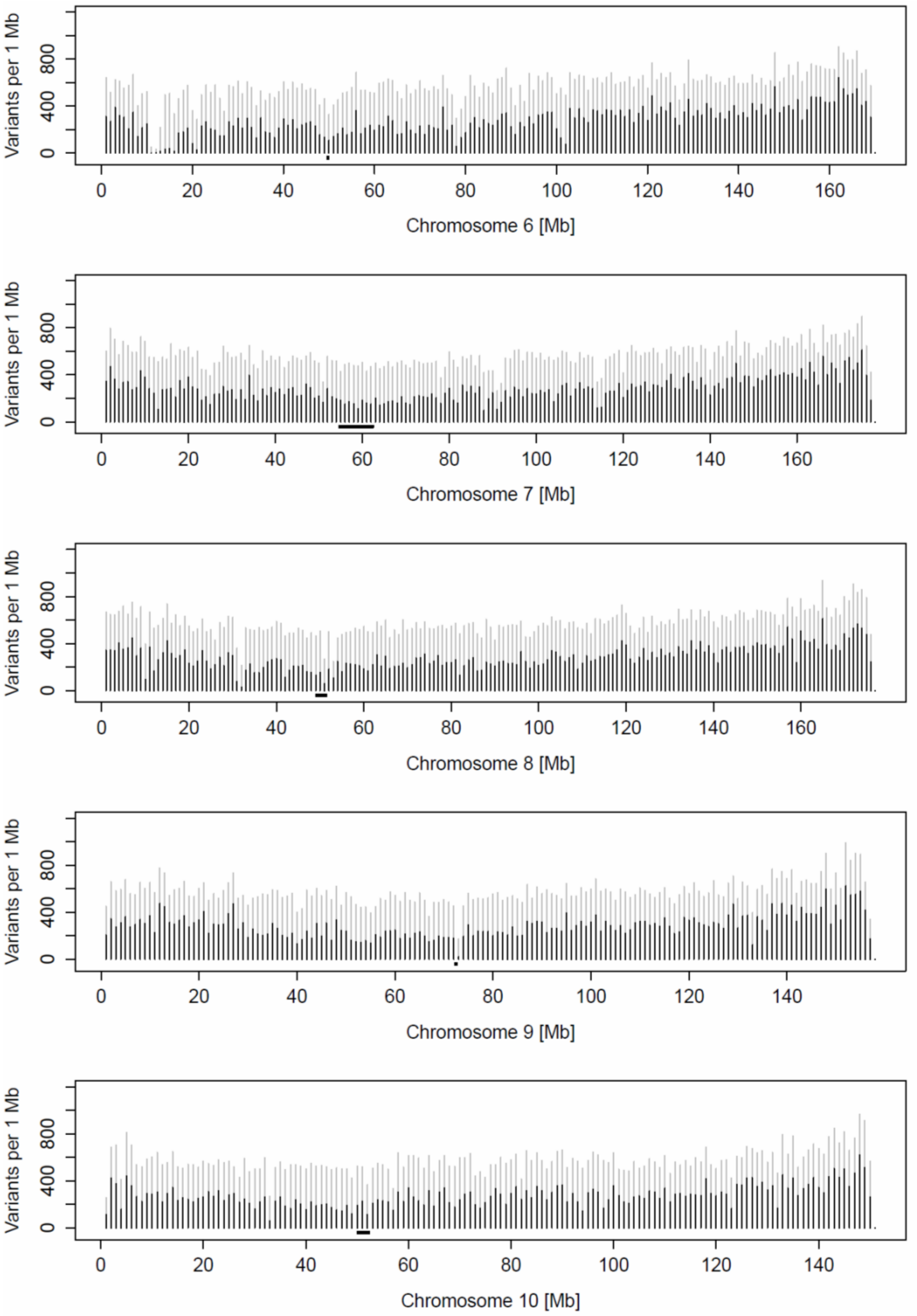

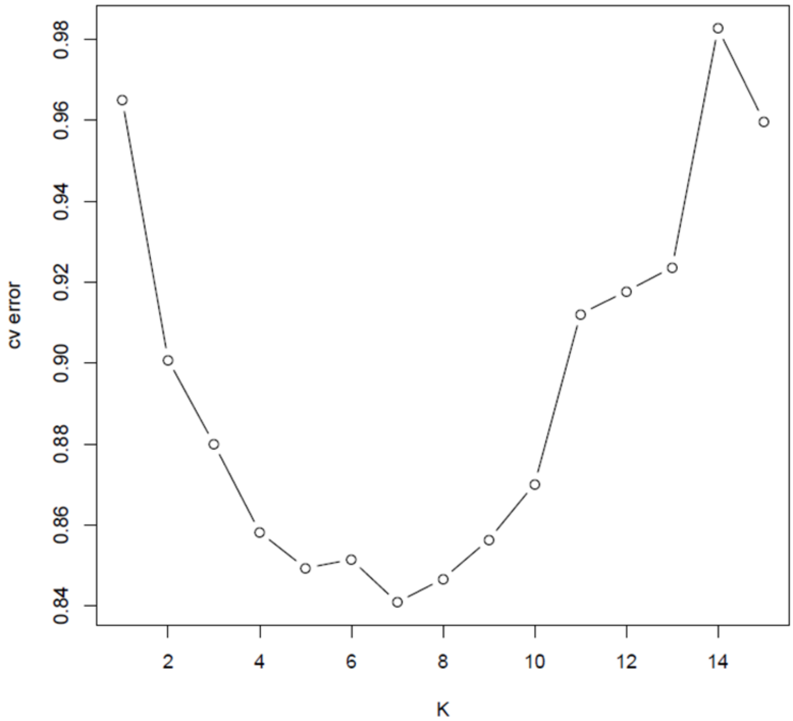

Figure S4: Cross-validation errors from ADMIXTURE for different values of K for 155 maize lines based on 251,152 variants including OTVs (markers in LD with $r^2 > 0.8$ were excluded).

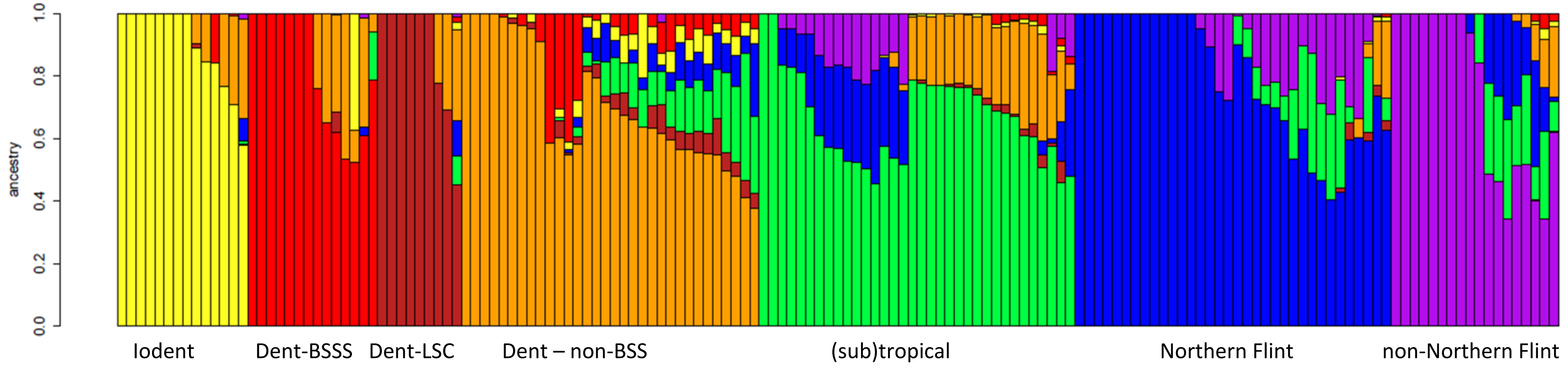

Figure S5: Subgroups identified in 155 maize lines of the validation panel as revealed by ADMIXTURE for K = 7 based on 251,152 variants including OTVs (markers in LD with $r^2 > 0.8$ were excluded).

Yellow: Iodents, red: BSSS Dents, brown: LSC, orange: non BSSS Dents, green: (sub)tropical lines or lines with ancestors of (sub)tropical origin, blue: Northern Flints, purple: non Northern Flints.

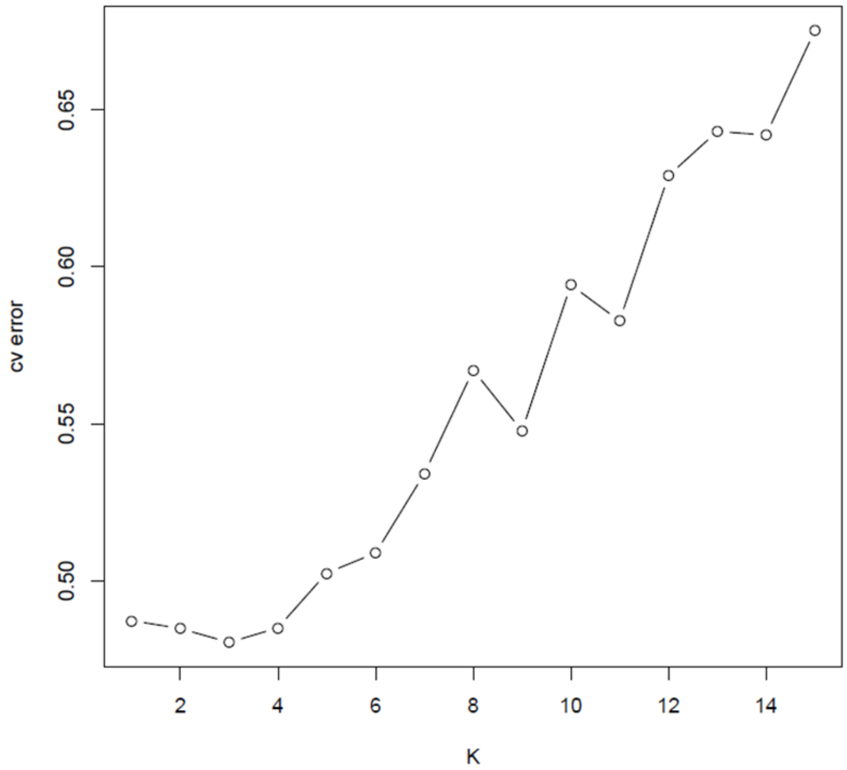

Figure S6: Cross-validation errors from ADMIXTURE for different values of K for 155 maize lines based on 27,099 flanking OTVs (markers in LD with r^2 > 0.8 were excluded).

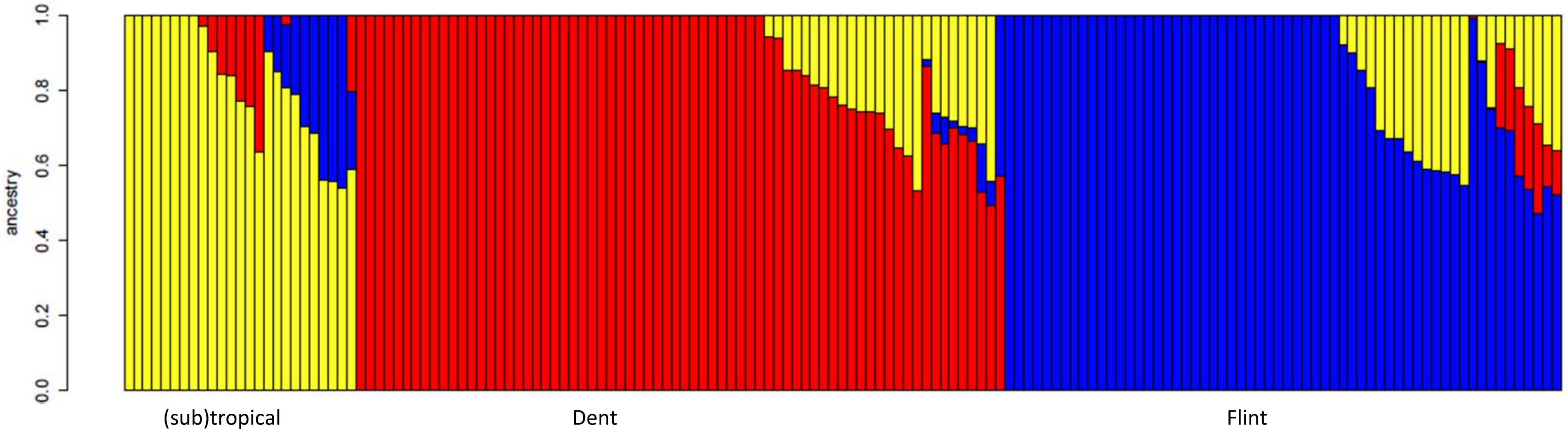

Figure S7: Subgroups identified in the 155 public lines of the validation panel as revealed by admixture for K = 3 based on 27,099 flanking OTVs (markers in LD with r^2 > 0.8 were excluded).

Yellow: (sub)tropical lines or lines with ancestors of (sub)tropical origin, red: Dent, blue: Flint.
